# Difference in spectral power density of sleep electroencephalography between individuals without insomnia and frequent hypnotic users with insomnia complaints

**DOI:** 10.1038/s41598-022-05378-6

**Published:** 2022-02-08

**Authors:** Jae Myeong Kang, Seo-Eun Cho, Jong Youn Moon, Soo In Kim, Jong Won Kim, Seung-Gul Kang

**Affiliations:** 1 Department of Psychiatry and Sleep Medicine Center, Gil Medical Center, Gachon University College of Medicine, 21, Namdong-daero 774 beon-gil, Namdong-gu, Incheon, 21565 Republic of Korea; 2 Department of Psychiatry, Gil Medical Center, Incheon, Republic of Korea; 3 Department of Preventive Medicine, Gil Medical Center, Gachon University College of Medicine, Incheon, Republic of Korea; 4 Department of Psychiatry, Ewha Womans University Mokdong Hospital, Ewha Womans University College of Medicine, Seoul, Republic of Korea; 5 Department of Healthcare IT, Inje University, Gimhae, Gyeongsangnam -do Republic of Korea

**Keywords:** Psychiatric disorders, Sleep

## Abstract

Previous spectral analysis studies on insomnia have shown inconsistent results due to their heterogeneity and small sample sizes. We compared the difference of electroencephalogram (EEG) spectral power during sleep among participants without insomnia, insomniacs with no hypnotic use, hypnotic users with no insomnia complaints, and hypnotic users with insomnia complaints using the Sleep Heart Health Study data, which is large sample size and has good quality control. The fast Fourier transformation was used to calculate the EEG power spectrum for total sleep duration within contiguous 30-s epochs of sleep. For 1985 participants, EEG spectral power was compared among the groups while adjusting for potential confounding factors that could affect sleep EEG. The power spectra during total sleep differed significantly among the groups in all frequency bands (*p*_corr_ < 0.001). We found that quantitative EEG spectral power in the beta and sigma bands of total sleep differed (*p*_corr_ < 0.001) between participants without insomnia and hypnotic users with insomnia complaints after controlling for potential confounders. The higher beta and sigma power were found in the hypnotic users with insomnia complaints than in the non-insomnia participants. This study suggests differences in the microstructures of polysomnography-derived sleep EEG between the two groups.

## Introduction

Insomnia is a common health problem characterized by difficulty in initiation and maintenance of sleep, and early awakening. About one third of the adult population reported insomnia symptoms as of 2006 and about 10 percent meet the criteria for insomnia disorder^1,2^. Recent studies have reported the increasing societal and economical costs of insomnia^3^.

Primary insomnia has been characterized by increased psychophysiological arousal and alterations of sleep continuity and architecture^4,5^. For instance, a previous meta-analysis of polysomnography (PSG) in insomnia noted disruption of sleep continuity and a significant reduction of rapid eye movement (REM) sleep and slow wave sleep in patients with insomnia compared to good sleepers^6^. However, contrary to expectations, there was no significant difference in PSG measures such as the proportion of REM sleep and slow wave sleep between participants with high and low insomnia severity index scores^7^. The patients with insomnia more than 5 years were not significantly different from the control group in terms of sleep latency, total sleep time, slow wave sleep, or REM ratio of PSG^6^. Additionally, similar sleep structures in insomnia and other mental disorders such as major depression might imply shared pathomechanisms^6,8,9^. Thus, alterations in sleep macrostructure measured by PSG are still insufficient to fully characterize primary insomnia.

The EEG recorded during sleep is a complex mixture of different frequency waveforms such as delta, theta, alpha, sigma, and beta^10^. Power spectral analysis is the most common method of quantitative EEG (qEEG) techniques and enables investigation of the microstructure of insomnia^8^. Many studies have been conducted on sleep qEEG of insomnia using spectral analysis. Previous studies have suggested that insomnia is related with physiological markers of hyperarousal during sleep^4,5^. In some studies, patients with insomnia demonstrated significantly elevated high spectral power values, including beta, sigma, or gamma EEG frequency, during NREM sleep compared to good sleepers^11,12^. However, other studies did not find this difference^13,14^. In healthy individuals, higher subjective sleep quality has been found to be related to decreased non-REM (NREM) sigma, but the effect sizes were small^15^. Thus, the findings regarding possible spectral power differences in healthy individuals and those with insomnia compared to good sleepers have been inconsistent. It has been argued that larger sample sizes are needed to overcome these inconsistencies and produce reliable research results^14^.

The clinical characteristics of the participants in those studies, such as the severity of insomnia and whether they were taking sleeping pills, also might have accounted for the discrepant results. The majority of patients with insomnia in the previous sleep EEG studies had mild insomnia and were not taking sleeping pills (i.e., hypnotics) or could stop the medication for at least two weeks^11–14,16,17^. However, many insomniacs are actually taking hypnotics^18^. Therefore, those studies might not reflect the clinical reality of insomnia patients. In addition, while there have been studies showing probable changes in sleep EEG due to the use of sleeping pills^19^, a large-scale study on the difference in sleep EEG between patients with insomnia who do and do not take sleeping pills is lacking.

This study investigated qEEG spectral power during total sleep time in a large, general population cohort, the Sleep Heart Health Study (SHHS), that ensured a large sample size with good quality control and a more representative assessment of sleep in the general population using PSG. The aims of this study were to compare the power spectral density of qEEG frequency bands 1) during total sleep time among various groups, including participants without insomnia (non-insomnia, NI), with insomnia and no hypnotic use (INH), using hypnotics with no insomnia complaints (HNI), and using hypnotics with insomnia complaints (HI); and 2) during NREM and REM sleep among the groups.

## Results

### Demographic characteristics and PSG results

**Figure 1:**
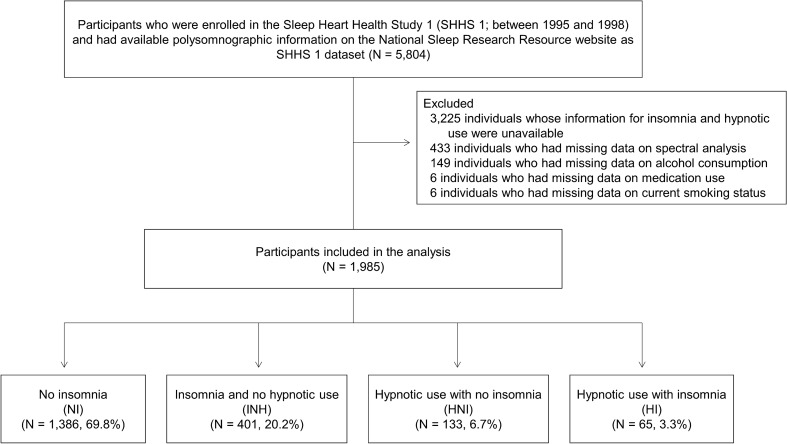
Flow chart of participant inclusion and exclusion.

Of the 5804 SHHS-1 participants with PSG data, we excluded 3225 because of missing data on insomnia and hypnotic use, 433 because of missing data on spectral analysis, 149 due to the lack of information on daily alcohol intake, 6 due to missing data on medication use (i.e., TCA, non-TCA, benzodiazepine), and 6 due to the lack of information on current smoking status. In total, 1985 participants met the inclusion criteria as described in the Materials and Methods section and were included in the analyses. Among the 1985 participants, 1386 (69.8%), 401 (20.2%), 133 (6.7%), and 65 (3.3%) were classified into the NI, INH, HNI, and HI groups, respectively. The flow chart of the inclusion and exclusion participants is presented in Fig. 1. The demographic characteristics of participants and their comparisons among groups are presented in Table 1. There were significant differences among the groups in terms of sex, with the highest proportion of female participants in the HI group. Significant differences were found in the amount of alcohol consumed per day and the recent use of medications, including benzodiazepine, TCA, and non-TCA, among the groups. As expected, the frequencies of each insomnia symptom were different among the groups.

**Table 1:** Comparison of demographic and clinical characteristics among the non-insomnia, insomniac with no hypnotic use, hypnotic user with no insomnia complaints, and hypnotic user with insomnia complaints groups.

Variables	NI (n = 1386)	INH (n = 401)	HNI (n = 133)	HI (n = 65)	Statistics
**Demographics**					
Age, years	63.5 ± 11.4	64.3 ± 10.9	65.5 ± 11.0	65.6 ± 11.1	*F* = 2.11, *p* = 0.097
Sex, female	609 (43.9%)	261 (65.1%)	86 (64.7%)	54 (83.1%)	*χ*^2^ = 96.17, *p* < 0.001
BMI, kg/m^2^	28.0 ± 5.1	28.1 ± 5.1	28.3 ± 5.6	28.6 ± 5.5	*F* = 0.37, *p* = 0.778
**Substance or medication**					
Current smoking	117 (8.4%)	43 (10.7%)	15 (11.3%)	6 (9.2%)	*χ*^2^ = 2.76, *p* = 0.430
Alcohol use per day	2.8 ± 5.4	2.6 ± 5.8	2.2 ± 4.7	1.2 ± 2.7	*F* = 13.28, *p* = 0.004
TCA use*	14 (1.0%)	10 (2.5%)	22 (16.5%)	11 (16.9%)	*χ*^2^ = 152.55, *p* < 0.001
Non-TCA use*	30 (2.2%)	20 (5.0%)	38 (28.6%)	5 (7.7%)	*χ*^2^ = 191.05, *p* < 0.001
Benzodiazepine use*	20 (1.4%)	43 (10.7%)	43 (32.3%)	28 (43.1%)	*χ*^2^ = 346.59, *p* < 0.001
**Insomnia symptoms**^**§**^					
Sleep initiation difficulty	0 (0%)	179 (44.6%)	0 (0%)	48 (73.8%)	*χ*^2^ = 1169.52, *p* < 0.001
Sleep maintenance difficulty	0 (0%)	226 (56.4%)	0 (0%)	33 (50.8%)	*χ*^2^ = 1504.44, *p* < 0.001
Early morning awakening	0 (0%)	181 (45.1%)	0 (0%)	27 (41.5%)	*χ*^2^ = 1229.41, *p* < 0.001
Taking sleeping pills	0 (0%)	0 (0%)	133 (100%)	65 (100%)	*χ*^2^ = 1846.00, *p* < 0.001
ESS score	7.5 ± 4.5	7.6 ± 4.7	7.2 ± 4.1	6.6 ± 4.8	*F* = 0.98, *p* = 0.402

Data are mean ± SD or number (percentage). Statistics were performed using ANOVA, Kruskal–Wallis, or Chi-square tests.

NI, non-insomnia; INH, insomniac with no hypnotic use; HNI, hypnotic user with no insomnia complaints; HI, hypnotic user with insomnia complaints; BMI, body mass index; TCA, tricyclic antidepressant; Non-TCA, non-tricyclic antidepressant; ESS, Epworth Sleepiness Scale.

*TCA, non-TCA, and benzodiazepine use: taking medication within two weeks.

^§^Presence (or absence) of each insomnia symptom was evaluated by the four questions below and those who reported 1: Never or 2: Rarely (1 × /month or less) were classified as having no insomnia, and those who responded almost always (16–30 × /month) were classified as having insomnia.

Question 1, sleep initiation difficulty: How often do you have trouble falling asleep?.

Question 2, sleep maintenance difficulty: How often do you wake up during the night and have difficulty resuming sleep?.

Question 3, early morning awakening: How often do you wake up too early in the morning and are unable to resume sleep?.

Question 4, taking sleeping pills: How often do you take sleeping pills or other medication to help you sleep?.

Table 2 shows the results of PSG and their comparisons among the four groups. The total sleep time and sleep efficiency were higher in the NI group than in the INH group and wake after sleep onset and REM sleep latency were lower in the NI group than in the other groups. The ratio of stage N2 and N3 differed among the groups. There was no significant difference in AHI among the groups.

**Table 2:** Polysomnographic findings and their comparisons among the groups.

Variables	NI (n = 1386)	INH (n = 401)	HNI (n = 133)	HI (n = 65)	ANOVA	
					Statistics	Significant difference after post-hoc analysis^¶^
**Sleep and wake time**						
Time in bed, min	434.2 ± 59.6	435.8 ± 60.3	429.9 ± 62.3	433.7 ± 70.3	*F* = 0.32, *p* = 0.813	
Total sleep time, min	364.7 ± 62.4	350.9 ± 68.5	357.7 ± 66.0	357.2 ± 66.4	*F* = 5.01, *p* = 0.002	NI vs. INH
Sleep efficiency, %	83.1 ± 9.7	79.5 ± 11.3	82.6 ± 9.6	81.3 ± 9.0	*F* = 8.34, *p* < 0.001	NI vs. INH INH vs. HNI
WASO, min	56.7 ± 42.5	68.4 ± 48.0	57.4 ± 39.6	60.6 ± 39.2	*F* = 7.62, *p* < 0.001	NI vs. INH INH vs. HNI
REM sleep latency, min	84.5 ± 54.1	92.4 ± 62.9	109.3 ± 73.6	116.3 ± 79.7	*F* = 11.85, *p* < 0.001	NI vs. INH NI vs. HNI NI vs. HI INH vs. HNI INH vs. HI
**Sleep stage, %**						
N1	5.3 ± 3.8	5.2 ± 3.8	5.8 ± 4.2	5.2 ± 3.5	*F* = 0.85, *p* = 0.467	
N2	56.7 ± 11.3	54.9 ± 12.1	55.8 ± 13.3	55.2 ± 12.2	*F* = 2.71, *p* = 0.044	NI vs. INH
N3	17.8 ± 11.7	19.9 ± 12.5	18.5 ± 12.3	21.6 ± 12.5	*F* = 2.71, *p* = 0.003	NI vs. INH NI vs. HI
R	20.2 ± 6.1	20.0 ± 6.7	19.8 ± 7.3	18.0 ± 6.8	*F* = 2.40, *p* = 0.066	
**Respiration**						
AHI, event per hour	14.9 ± 15.1	14.2 ± 14.9	13.0 ± 15.8	10.6 ± 12.2	*F* = 2.33, *p* = 0.073	
Arousal index	19.4 ± 10.8	18.8 ± 11.0	19.4 ± 11.9	18.0 ± 8.8	*F* = 0.52, *p* = 0.672	

Data are mean ± SD. Statistics were performed using analysis of variance.

NI, non-insomnia; INH, insomniac with no hypnotic use; HNI, hypnotic user with no insomnia complaints; HI, hypnotic user with insomnia complaints; ANOVA, analysis of variance; WASO, wake after sleep onset; REM, rapid eye movement; N1, stage 1 non-rapid eye movement sleep; N2, stage 2 non-rapid eye movement sleep; N3, stage 3 non-rapid eye movement sleep; R, rapid eye movement sleep; AHI, apnea–hypopnea index.

^¶^The post-hoc analysis was performed using Bonferroni test.

### Comparison of absolute spectral EEG power among groups

**Figure 2:**
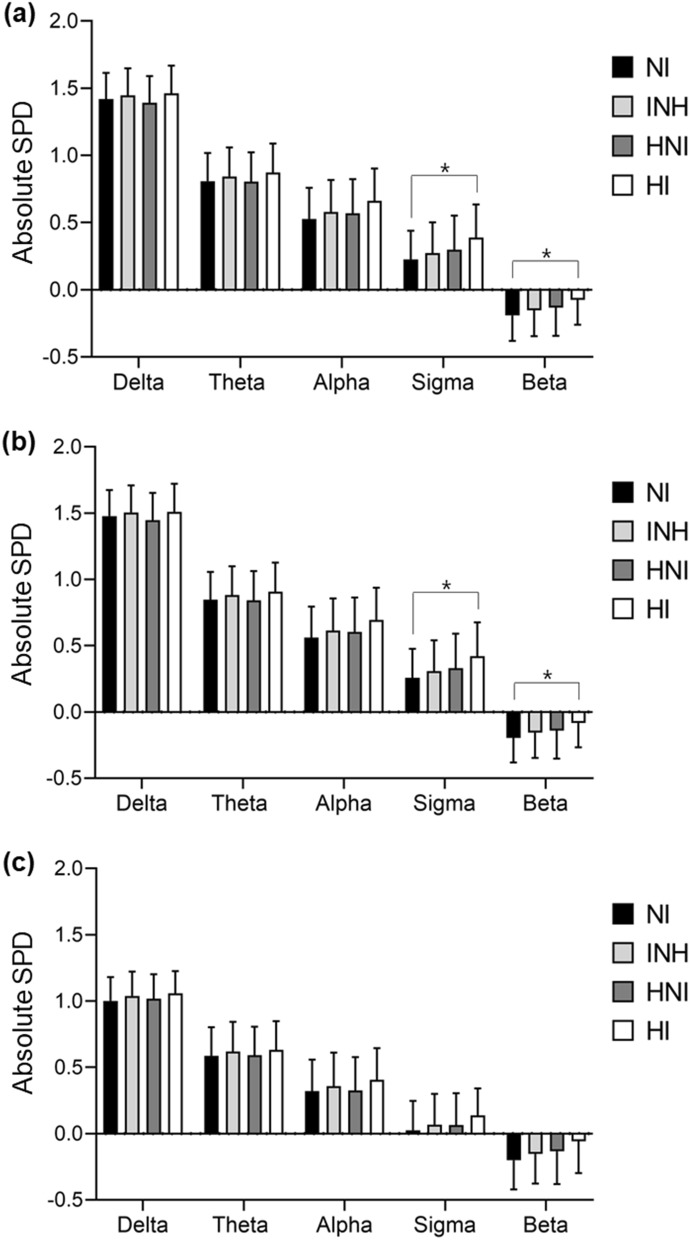
Comparisons of the absolute spectral power density during (**a**) total, (**b**) NREM, and (**c**) REM sleep among the groups. Log-transformed absolute spectral power density (Y-axis) during (**a**) total, (**b**) NREM, and (**c**) REM sleep for each EEG frequency band (X-axis): delta (1–4 Hz), theta (4–8 Hz), alpha (8–12 Hz), sigma (12–15 Hz), and beta (15–20 Hz). The mean of absolute spectral power density during total, NREM, and REM sleep among the groups is plotted as the height of the column. Error bars represent the upper standard deviations. We drew the lines between the combinations that differed significantly by ANCOVA and marked them with an asterisk (*). NREM, non-rapid eye movement sleep; REM, rapid eye movement sleep; NI, non-insomnia; INH, insomnia with no hypnotic use; HNI, hypnotic user with no insomnia complaints; HI, hypnotic user with insomnia complaints; SPD, spectral power density; ANCOVA, analysis of covariance.

Table 3 and Fig. 2a provide the comparisons of the absolute spectral power in central electrodes during total sleep among the NI, INH, HNI, and HI groups. There were significant differences in the spectral power in all frequency bands among the groups for the ANOVA tests (*p* corrected < 0.05 in all frequency bands). After controlling for potential confounders (i.e., age, sex, AHI, current smoking status, usual alcohol intake per day, and recent use of TCA, non-TCA, and benzodiazepine) using an ANCOVA, there were significant differences in the sigma (12–15 Hz; *F* = 4.35, *p* = 0.005, *p* corrected = 0.023) and beta (15–20 Hz; *F* = 4.80, *p* = 0.002, *p* corrected = 0.012) frequency bands among the groups. In the post-hoc analysis, the absolute spectral power in the beta and sigma bands during total sleep were higher in the HI group than in the NI group.

**Table 3:** Comparison of the absolute spectral power density^§^ during total sleep among the groups after controlling for potential confounding factors.

Spectral bands	NI (n = 1386)	INH (n = 401)	HNI (n = 133)	HI (n = 65)	ANOVA	ANCOVA*	
					Statistics	Statistics	Significant difference after post-hoc analysis^¶^
Delta (1–4 Hz)	1.419 ± 0.195	1.447 ± 0.201	1.391 ± 0.200	1.462 ± 0.207	*F* = 4.13, *p* = 0.006, ***p*** ***corr* = 0.031**	*F* = 0.98, *p* = 0.401, *p* *corr* > 0.999	None
Theta (4–8 Hz)	0.808 ± 0.210	0.843 ± 0.216	0.804 ± 0.219	0.872 ± 0.216	*F* = 4.42, *p* = 0.004, ***p*** ***corr* = 0.021**	*F* = 0.616, *p* = 0.605, *p* *corr* > 0.999	None
Alpha (8–12 Hz)	0.525 ± 0.233	0.578 ± 0.239	0.567 ± 0.256	0.661 ± 0.241	*F* = 11.31, *p* < 0.001, ***p*** ***corr* < 0.001**	*F* = 1.58, *p* = 0.192, *p* *corr* = 0.958	None
Sigma (12–15 Hz)	0.224 ± 0.214	0.273 ± 0.228	0.297 ± 0.254	0.387 ± 0.247	*F* = 17.81, *p* < 0.001, ***p*** ***corr* = 0.001**	*F* = 4.35, *p* = 0.005, ***p*** ***corr* = 0.023**	**NI** **vs.** **HI**
Beta (15–20 Hz)	− 0.192 ± 0.190	− 0.153 ± 0.193	− 0.133 ± 0.211	− 0.076 ± 0.185	*F* = 13.34, *p* < 0.001, ***p*** ***corr* < 0.001**	*F* = 4.80, *p* = 0.002, ***p*** ***corr* = 0.012**	**NI** **vs.** **HI**

Data are mean ± SD.

NI, non-insomnia; INH, insomniac with no hypnotic use; HNI, hypnotic user with no insomnia complaints; HI, hypnotic user with insomnia complaints; ANOVA, analysis of variance; ANCOVA, analysis of covariance; TCA, tricyclic antidepressant.

^**§**^log-transformed absolute spectral power density (log_10_ μV^2^); *ANCOVA controlling for age, sex, apnea–hypopnea index, current smoking status, usual alcohol intake per day, and recent use of TCA, non-TCA, and benzodiazepine; *p corr*, *p* value after Bonferroni correction (uncorrected *p* value × 5) for correction of multiple comparisons. Values in bold indicate significance after Bonferroni correction (*p* < 0.05). ^*¶*^The post-hoc analysis was performed using Bonferroni test.

Table 4 and Fig. 2b and c present the results for comparisons of the absolute spectral power in central electrodes during NREM and REM sleep among the groups. There were significant differences (*p* corrected < 0.05) in the spectral power in all frequency bands except the delta band during NREM sleep (*p* corrected = 0.085) and in all frequency bands except the theta band during REM sleep (*p* corrected = 0.323) among the groups in the ANOVA tests. There were also significant differences in the sigma (*p* corrected = 0.030) and beta (*p* corrected = 0.020) bands during NREM sleep after controlling for potential confounders, with a higher power density in the HI group than in the NI group in the post-hoc analysis. During REM sleep, a higher power density in the beta band in the NI group than in the HI group was found in the post-hoc analysis of the ANCOVA test but failed to survive the correction for multiple comparisons (*p* corrected = 0.075). The propensity score-matched analysis also confirmed the higher power density of sigma and beta power in the HI group than in the NI group during total and NREM sleep. The demographic and clinical characteristics (Table S1), polysomnographic findings (Table S2), and the comparison of the spectral power density between the groups (Table S3 and S4) are shown in the supplemental data.

**Table 4:** Comparison of the absolute spectral power density^§^ during NREM and REM sleep among the groups after controlling for potential confounding factors.

Spectral bands	NI (n = 1386)	INH (n = 401)	HNI (n = 133)	HI (n = 65)	ANOVA	ANCOVA*	
					Statistics	Statistics	Significant difference after post-hoc analysis^¶^
**NREM**							
Delta (1–4 Hz)	1.478 ± 0.197	1.505 ± 0.205	1.446 ± 0.207	1.511 ± 0.211	*F* = 3.91, *p* = 0.008, *p* *corr* = 0.085	*F* = 0.76, *p* = 0.517, *p* *corr* > 0.999	None
Theta (4–8 Hz)	0.846 ± 0.210	0.882 ± 0.217	0.841 ± 0.222	0.907 ± 0.220	*F* = 4.39, *p* = 0.004, ***p*** ***corr* = 0.043**	*F* = 0.62, *p* = 0.603, *p* *corr* > 0.999	None
Alpha (8–12 Hz)	0.560 ± 0.234	0.614 ± 0.242	0.603 ± 0.260	0.694 ± 0.244	*F* = 11.23, *p* < 0.001, ***p*** ***corr* < 0.001**	*F* = 1.70, *p* = 0.165, *p* *corr* > 0.999	None
Sigma (12–15 Hz)	0.258 ± 0.218	0.308 ± 0.232	0.330 ± 0.260	0.420 ± 0.256	*F* = 17.07, *p* < 0.001, ***p*** ***corr* < 0.001**	*F* = 4.63, *p* = 0.003, ***p*** ***corr* = 0.030**	**NI** **vs.** **HI**
Beta (15–20 Hz)	− 0.196 ± 0.186	− 0.157 ± 0.191	− 0.141 ± 0.212	− 0.085 ± 0.183	*F* = 12.32, *p* < 0.001, ***p*** ***corr* < 0.001**	*F* = 4.93, *p* = 0.002, ***p*** ***corr* = 0.020**	**NI** **vs.** **HI**
**REM**							
Delta (1–4 Hz)	1.002 ± 0.179	1.039 ± 0.183	1.018 ± 0.184	1.058 ± 0.168	*F* = 5.74, *p* = 0.001, ***p*** ***corr* = 0.007**	*F* = 1.52, *p* = 0.206, *p* *corr* > 0.999	None
Theta (4–8 Hz)	0.585 ± 0.217	0.618 ± 0.226	0.590 ± 0.216	0.632 ± 0.217	*F* = 2.93, *p* = 0.032, *p* *corr* = 0.323	*F* = 0.17, *p* = 0.918, *p* *corr* > 0.999	None
Alpha (8–12 Hz)	0.319 ± 0.239	0.357 ± 0.253	0.326 ± 0.250	0.405 ± 0.239	*F* = 4.50, *p* = 0.004, ***p*** ***corr* = 0.038**	*F* = 0.60, *p* = 0.616, *p* *corr* > 0.999	None
Sigma (12–15 Hz)	0.025 ± 0.221	0.066 ± 0.233	0.064 ± 0.240	0.137 ± 0.204	*F* = 8.09, *p* < 0.001, ***p*** ***corr* < 0.001**	*F* = 1.13, *p* = 0.335, *p* *corr* > 0.999	None
Beta (15–20 Hz)	− 0.202 ± 0.220	− 0.154 ± 0.224	− 0.134 ± 0.247	− 0.058 ± 0.241	*F* = 13.91, *p* < 0.001, ***p*** ***corr* < 0.001**	*F* = 3.99, *p* = 0.008, *p* *corr* = 0.075	NI vs. HI

Data are mean ± SD.

Abbreviations: NI, non-insomnia; INH, insomniac with no hypnotic use; HNI, hypnotic user with no insomnia complaints; HI, hypnotic user with insomnia complaints; ANOVA, analysis of variance; ANCOVA, analysis of covariance; NREM, non-rapid eye movement sleep; REM, rapid eye movement sleep; TCA, tricyclic antidepressant.

^**§**^log-transformed absolute spectral power density (log_10_ μV^2^); *ANCOVA controlling for age, sex, apnea–hypopnea index, current smoking status, usual alcohol intake per day, and recent use of TCA, non-TCA, and benzodiazepine; *p corr*, *p* value after Bonferroni correction (uncorrected *p* value × 10) for correction of multiple comparisons. Values in bold indicate significance after Bonferroni correction (*p* < 0.05). ^*¶*^ The post-hoc analysis was performed using Bonferroni test.

## Discussion

This study aimed to investigate the difference of spectral power density of sleep EEG derived from PSG in a large sample of the general population that included individuals who differed in insomnia status. The results showed spectral power differences in the beta and sigma EEG frequency bands during total sleep among the groups, with higher activity in the HI group compared to the NI group after controlling for potential confounders.

Regarding the spectral power bands, the spectral power of the beta and sigma bands, which are high-frequency bands, were significantly increased in the HI group compared to the NI group. Several previous studies on insomnia reported increased spectral power of the high-frequency bands in qEEG and interpreted it as a hyperarousal marker of insomnia^12,20^. Conversely, other studies did not show a difference in spectral power between insomnia and control groups, or showed significant differences only in specific subgroups or subtypes of insomnia^14,21,22^. One study reported increased high-frequency and low-frequency EEG activity during early NREM periods only in women^14^, and another study reported greater alpha, sigma, and beta EEG activity and lower delta during NREM sleep in patients with subjective insomnia than in those who were good sleepers, but these differences were not found when comparing those with objective insomnia to good sleepers^21^. Another study reported the lower levels in the 18–29.75 Hz frequency range (‘beta 2’) in sleep onset insomnia and suggested that mechanisms other than hyperarousal may be involved in the etiology of sleep onset insomnia^22^. Although no sleep EEG differences between patients with insomnia and good sleepers were reported in several recent, relatively large studies (803 participants with insomnia and 811 controls^23^; 50 participants with insomnia and 32 controls^13^), insomnia patients with hypnotic use were excluded in those studies. Thus, the higher activity of sigma and beta power in the HI group than in the NI group in this study, taken from a large sample of the general population, might be indicative of the microstructural signature of insomnia symptoms including sleep initiation and maintenance and early morning awakening.

The results showed that the HI group, those who experience insomnia even with hypnotics, had significantly higher spectral power in beta and sigma bands than that in the NI group (i.e., good sleepers), but activity in these bands did not significantly differ from that in the INH or HNI groups after controlling for potential confounders. These results might imply that the spectral power density during sleep is more affected by the severity of insomnia than by the use of hypnotics, which seems inconsistent with previous studies^24–26^. Benzodiazepine and zolpidem have been reported to be associated with an increased power in high-frequency EEG bands and a decreased power in low-frequency EEG bands^24–27^. In a previous polysomnographic study, spectral power density in NREM sleep was reduced in the low-frequency range (1.25–2.5 Hz; 5.25–10.0 Hz) and increased in the spindle frequency range (12.25–13.0 Hz) after the administration of zolpidem^25^. Another study performed on adults over 46 years old compared the spectral power density between chronic benzodiazepine users with insomnia, drug-free insomnia participants, and good sleepers, and showed that benzodiazepine users exhibited significantly less delta and theta activity over the night and more beta1 (14.04–21.84) activity within third NREM-REM sleep cycle (cycle 3) than did good sleepers^26^. When compared to drug-free insomnia participants, benzodiazepine users had less delta and theta activity within cycle 2 only, and more beta1 activity within cycle 4^26^. In this study, that a difference was found only between the HI and NI groups may imply the impression that hypnotic use has less an effect on sleep EEG than we had expected. Regarding this, we believe that the large sample (*n* = 1985) drawn from the general population might have affected these results compared to the small clinical samples of previous studies [number of total participants: 8^25^, 30^22^, 47^26^, and 50^21^]. In addition, the mixture of different kinds of hypnotic medications that participants could have been taking given the general question as to whether they “take sleeping pills or other medication to help you sleep” might have weakened the effect of hypnotics on the results. However, after adjusting for potential sociodemographic and clinical confounders (i.e., age, sex, AHI, current smoking status, usual alcohol intake per day, recent use of TCA, non-TCA, and benzodiazepine) and incorporating subtypes of insomnia symptoms (initiation, maintenance, and early morning awakening), it appears that the high-frequency spectral power bands may also clarify the microstructure of insomnia.

The results of spectral power comparisons among the groups showed different patterns in NREM and REM sleep. In NREM sleep, there were significant differences of beta and sigma activity between the HI and NI groups while only beta activity showed a group difference in REM sleep that failed to survive a correction for multiple comparisons. These results are in line with previous studies reporting more spectral power changes in NREM sleep than in REM sleep^13,14,21,23^. Due to the eye movements, which are a prominent feature of the REM period, spectral power density during REM sleep is substantially distorted and limited in REM sleep quantitative studies. However, a decreased proportion of REM sleep in insomnia patients has been found in previous studies^17,28,29^ and also in our study. As REM sleep-related processes can contribute to disturbed sleep perception in patients with insomnia^29^, increased beta activity in the REM period in this study, even after considering confounders in a large population, might explain the pathomechanism of the insomnia symptoms.

There are several limitations to this study. Since the SHHS was designed to investigate the association between sleep-disordered breathing and cardiovascular diseases^30^, we only included adults aged over 40 years; habitual snorers were likely oversampled^30^. Therefore, the participants in our study were relatively old (age: 63.8 ± 11.3 years) with high AHI and ESS scores. In addition, the sample sizes in each group were unbalanced, although we used the propensity score matching method to overcome this problem. Hence, the results of this study cannot be generalized to other populations. The lack of information on the type and half-life of benzodiazepines and the dosage of hypnotics used could bias the results of the EEG activity. Additionally, the classification of the presence of insomnia used in this study (prevailing insomnia symptoms more than 15 days per month) might have resulted in different findings from what would be observed in a clinical setting.

The advantage of this study is that it was possible to demonstrate the true power difference among the groups by controlling for confounding factors. In contrast to previous studies that excluded patients with insomnia who were taking medications that can affect sleep, this cohort included patients with severe insomnia who were taking sleeping pills, which can elucidate sleep EEG in providing a more comprehensive understanding along the insomnia continuum. Another advantage of this study is the significantly larger sample size compared to previous spectral analysis studies on insomnia. It is thought that the power spectral findings on insomnia that were not confirmed in some of the other studies due to limitations of those studies (i.e., different spectral analysis methods, different characteristics of insomnia patients, and a small number of participants) could be established to some extent through the results of this study.

## Conclusions

To the best of our knowledge, this is the largest spectral power comparison among non-insomnia participants and hypnotic users with and without insomnia to date, highlighting the importance of these findings. In summary, we found that qEEG spectral power in the beta and sigma bands during sleep differ between people without insomnia and those with insomnia who take hypnotics, with higher levels of both bands found in the latter group. This study suggests differences in the microstructures of PSG-derived sleep EEG among those without insomnia and those with insomnia who take hypnotics.

## Materials and methods

### Data sources and study cohort

The SHHS is a large, multi-center, community-based, prospective cohort study that sought to determine the cardiovascular and other consequences of sleep-disordered breathing (ClinicalTrials.gov Identifier: NCT00005275)^30^. From 1995 to 1998, the participants older than 40 years underwent unattended PSG and structured health interviews and completed sleep questionnaires. The design and aims of the study have been previously reported^30^. The data collected included information on use of psychotropic medications (i.e., benzodiazepine, tricyclic antidepressants [TCA], and non-TCA within two weeks) and substances (i.e., current smoking status and amount of alcohol use per day) that might affect sleep EEG, in addition to age and sex.

Also available were data regarding frequency of insomnia and hypnotic use that were evaluated using four items [(1) “Have trouble falling asleep,” (2) “Wake up during the night and have difficulty getting back to sleep,” (3) “Wake up too early in the morning and be unable to get back to sleep,” and (4) “Take sleeping pills or other medication to help you sleep”] that were scored on a 5-point Likert scale (1, never; 2, rarely [1 ×/month or less]; 3, sometimes [2–4 ×/month]; 4, often [5–15 ×/month]; 5, almost always [16–30 ×/month]). Participants were defined as having insomnia if they answered with a ‘5’ to any of the four items. Using these questions, we divided the participants into four groups: NI, INH, HNI, and HI. The NI group answered with a 1 or 2 to all questions; the INH group answered with a 5 to one or more of questions 1–3, but answered with a 1 or 2 to question 4; the HNI group answered with a 5 to question 4, but answered with a 1 or 2 to questions 1–3; and the HI group answered with a 5 to at least one of questions 1–3 and a 5 to question 4. The participants in the HI group were those who still had insomnia symptoms despite hypnotics usage, and the participants in the HNI group were those who resolved their insomnia symptoms with hypnotics. Participants who did not fit any of the criteria were excluded from the analyses. Additionally, only participants who had complete data for spectral analysis including covariates (i.e., age, sex, apnea–hypopnea index [AHI], TCA use, non-TCA use, benzodiazepine use, current smoking status, and usual alcohol intake per day) were included in the analyses.

We had access to the SHHS-1 (first round of PSG recording of SHHS) database from the National Sleep Research Resource website (https://sleepdata.org/datasets/shhs) by acquiring a signed agreement with Brigham and Women’s Hospital; our project was exempted from review by the institutional review board (IRB No. GDIRB2018-005) of Gil Medical Center.

### Polysomnography

All participants underwent unattended, in-home, overnight PSG as previously described using a Compumedics P-series recording system (Compumedics)^31^. The recording montage included a C3-A2 and C4-A1 EEG, left and right electrooculograms, chin electromyogram, single-lead electrocardiogram, airflow by an oral-nasal thermistor, oxyhemoglobin saturation by pulse oximetry, measurement of thoracic and abdominal effort by impedance plethysmography, and body position by mercury strain gauge. Sleep stage scoring of all nocturnal recordings was conducted by trained technicians using the Rechtschaffen and Kales criteria^32^ at a centralized reading center. These data were defined and scored using the AHI in various ways; among them, we chose the recommended hypopnea criteria from the American Academy of Sleep Medicine manual^33^.

### Spectral analysis

The spectral analysis method used was described in a previous study comparing the sleep EEG between smokers and nonsmokers^34^. The C3-A2 and C4-A1 EEG recordings were sampled at 125 Hz and analyzed using the fast Fourier transform. The fast Fourier transform was conducted on a 5-s EEG segment to obtain a frequency resolution of 0.2 Hz. Each 5-s EEG segment was first windowed with a Hanning tapering window prior to computing the power spectra. The power content expressed as μV^2^ for each 30-s epoch of sleep was determined as the average power across the six 5-s segments of the EEG. Power spectra were computed for each EEG frequency band: delta (1–4 Hz), theta (4–8 Hz), alpha (8–12 Hz), sigma (12–15 Hz), and beta (15–20 Hz). To control for low-frequency artifacts, such as sweating and respiration, frequencies < 0.8 Hz were excluded from analyses^34^. For the present analysis, data derived from the central EEG electrodes (i.e., [C3/A2 + C4/A1]/2) recorded over the total sleep period were used. For analysis, we used the absolute spectral power, which is the integral of all the power values within each frequency range. To achieve normal distributions, all absolute power data were log transformed^35^.

### Statistical analysis

Chi-square test was used for categorical variables, and Kruskal–Wallis test, analysis of variance (ANOVA), and analysis of covariance (ANCOVA) were used to compare the demography, PSG characteristics, and absolute spectral EEG power among the groups. The ANCOVA was performed to compare the power in each spectral bandwidth among groups after controlling for potential confounders including age and sex. The significance in comparison of the spectral power during total sleep among the groups was defined as *p* < 0.05 after Bonferroni correction, which was calculated as the uncorrected *p* value × 5 (5 being the number of EEG frequency bands). The significance in comparison of the spectral power during NREM and REM sleep among the groups was defined as *p* < 0.05 after Bonferroni correction, which was calculated as the uncorrected *p* value × 10. IBM SPSS software (version 25.0, IBM Corp, Armonk, NY, USA) was used for data analysis. After conducting group comparisons in all participants, we performed an additional analysis by sampling the control group using a propensity score matching method based on logistic regression^36^ using the ‘Matchit’ package in R (http://cran.r-project.org). In propensity score matching, a total of 325 NI participants were selected in a 1:5 ratio to HI participants with nearest neighbor matching. Propensity scores were calculated using age group (< 50, 50–59, 60–69, and ≥ 70 years), sex, AHI (≤ 15 and > 15), alcohol consumption, smoking status, and medication status (TCA, non-TCA, and benzodiazepine).

## Supplementary Information

Supplementary Tables.

## Data Availability

The datasets used in this study are publicly available at the Sleep Heart Health Study website (https://sleepdata.org/datasets/shhs).
