# Supplementary material for: Difference in spectral power density of sleep electroencephalography between individuals without insomnia and frequent hypnotic users with insomnia complaints
Source: Sci Rep. 2022 Feb 8;12:2117. doi: 10.1038/s41598-022-05378-6 (PMC8826925; doi:10.1038/s41598-022-05378-6)
Supplement: Supplementary file 1 — Supplementary Tables. [file 41598_2022_5378_MOESM1_ESM.docx]

**Difference in spectral power density of sleep electroencephalography between individuals without insomnia and frequent hypnotic users with insomnia complaints**

Jae Myeong Kang ^1,*^, Seo-Eun Cho ^2,*^, Jong Youn Moon ^3^,

Soo In Kim ^4^, Jong Won Kim ^5^, Seung-Gul Kang ^1,†^

**Supplemental data**

| **Table S1**. Demographic and clinical characteristics in the non-insomnia and hypnotic user with insomnia complaints groups: propensity matched results | | | | |
| --- | --- | --- | --- | --- |
| *Variables* | | *NI (n=325)* | *HI (n=65)* | *Statistics* |
| *Demographics* | |  |  |  |
|  | Age, years | 66.0±11.1 | 65.6±11.1 | *Z*=–0.16, *p*=0.874 |
|  | Sex, female | 295 (90.8%) | 54 (83.1%) | *χ*^2^=3.41, *p*=0.065 |
| *Substance or medication* | |  |  |  |
|  | Current smoking | 37 (11.4%) | 6 (9.2%) | *χ*^2^=0.27, *p*=0.613 |
|  | Alcohol use per day | 1.3±3.3 | 1.2±2.7 | *Z*=–0.76, *p*=0.450 |
|  | TCA use* | 14 (4.3%) | 11 (16.9%) | *χ*^2^=14.37, *p*<0.001 |
|  | Non-TCA use* | 28 (8.6%) | 5 (7.7%) | *χ*^2^=0.06, *p*=0.807 |
|  | Benzodiazepine use* | 20 (6.2%) | 28 (43.1%) | *χ*^2^=68.42, *p*<0.001 |
| *Insomnia symptoms* ^§^ | |  |  |  |
|  | Sleep initiation difficulty | 0 (0%) | 48 (73.8%) | *χ*^2^=273.68, *p*<0.001 |
|  | Sleep maintenance difficulty | 0 (0%) | 33 (50.8%) | *χ*^2^=180.25, *p*<0.001 |
|  | Early morning awakening | 0 (0%) | 27 (41.5%) | *χ*^2^=145.04, *p*<0.001 |
|  | Taking sleeping pills | 0 (0%) | 65 (100%) | *χ*^2^=390.00, *p*<0.001 |
| ESS score | | 7.3±4.1 | 6.6±4.8 | *Z*=–1.33, *p*=0.183 |
| Data are mean±SD or number (percentage)  Statistics were performed using Mann Whitney-U or Chi-square tests.  Propensity scores were calculated using age group (<50, 50–59, 60–69, and ≥70 years), sex, apnea-hypopnea index (≤15 and >15), alcohol consumption, smoking status, and recent use of medications (TCA, non-TCA, and benzodiazepine).  *TCA, non-TCA, and benzodiazepine use: taking medication within two weeks.  ^§^ Presence (or absence) of each insomnia symptom was evaluated by the four questions below and those who reported 1: Never or 2: Rarely (1 × /month or less) were classified as having no insomnia, and those who responded almost always (16–30 × /month) were classified as having insomnia.  Question 1, sleep initiation difficulty: How often do you have trouble falling asleep?  Question 2, sleep maintenance difficulty: How often do you wake up during the night and have difficulty resuming sleep?  Question 3, early morning awakening: How often do you wake up too early in the morning and are unable to resume sleep?  Question 4, taking sleeping pills: How often do you take sleeping pills or other medication to help you sleep?  Abbreviations: NI, non-insomnia; INH, insomniac with no hypnotic use; HNI, hypnotic user with no insomnia complaints; HI, hypnotic user with insomnia complaints; TCA, tricyclic antidepressant; Non-TCA, non-tricyclic antidepressant; ESS, Epworth Sleepiness Scale | | | | |

| **Table S2**. Polysomnographic findings in the non-insomnia and hypnotic user with insomnia complaints groups: propensity matched results | | | | |
| --- | --- | --- | --- | --- |
| *Variables* | | *NI (n=325)* | *HI (n=65)* | *Statistics* |
| *Sleep and wake time* | |  |  |  |
|  | Time in bed, min | 435.8±60.9 | 433.7±70.3 | *Z*=–0.37, *p*=0.712 |
|  | Total sleep time, min | 369.5±64.6 | 357.2±66.4 | *Z*=–1.45, *p*=0.147 |
|  | Sleep efficiency, % | 83.2±10.3 | 81.3±9.0 | *Z*=–1.72, *p=*0.086 |
|  | WASO, min | 53.7±38.8 | 60.6±39.2 | *Z*=–1.66, *p=*0.098 |
|  | REM sleep latency, min | 95.8±65.7 | 116.3±79.7 | *Z*=–2.01, *p=*0.045 |
| *Sleep stage, %* | |  |  |  |
|  | N1 | 4.4±3.2 | 5.2±3.5 | *Z*=–1.44, *p*=0.149 |
|  | N2 | 53.0±11.9 | 55.2±12.2 | *Z*=–1.29, *p*=0.196 |
|  | N3 | 22.9±12.3 | 21.6±12.5 | *Z*=–0.49, *p*=0.623 |
|  | R | 19.7±6.7 | 18.0±6.8 | *Z*=–1.68, *p*=0.093 |
| *Respiration* | |  |  |  |
|  | AHI, event per hour | 8.5±10.1 | 10.6±12.2 | *Z*=–1.30, *p*=0.195 |
| Arousal index | | 16.6±8.8 | 18.0±8.8 | *Z*=–1.43, *p*=0.153 |
| Data are mean±SD; Statistics were performed using Mann Whitney-U test.  Propensity scores were calculated using age group (<50, 50–59, 60–69, and ≥70 years), sex, apnea-hypopnea index (≤15 and >15), alcohol consumption, smoking status, and recent use of medications (TCA, non-TCA, and benzodiazepine).  Abbreviations: NI, non-insomnia; INH, insomniac with no hypnotic use; HNI, hypnotic user with no insomnia complaints; HI, hypnotic user with insomnia complaints; ANOVA, analysis of variance; WASO, wake after sleep onset; REM, rapid eye movement; N1, stage 1 non-rapid eye movement sleep; N2, stage 2 non-rapid eye movement sleep; N3, stage 3 non-rapid eye movement sleep; R, rapid eye movement sleep; AHI, apnea-hypopnea index; TCA, tricyclic antidepressant; Non-TCA, non-tricyclic antidepressant | | | | |

| **Table S3.** Comparisons of the absolute spectral power density^§^ during total sleep between the NI and HI groups before and after propensity score matching | | | | | | | |
| --- | --- | --- | --- | --- | --- | --- | --- |
| *Spectral bands* | *Before propensity score matching* | | |  | *After propensity score matching* | | |
|  | *NI (n=1386)* | *HI (n=65)* | *Statistics* |  | *NI (n=325)* | *HI (n=65)* | *Statistics* |
| Delta  (1–4 Hz) | 1.419 ±0.195 | 1.462 ±0.207 | *Z*=–1.61, *p*=0.107,  *p corr*=0.535 |  | 1.492 ±0.186 | 1.462 ±0.207 | *Z*=–1.13, *p*=0.260,  *p corr*>0.999 |
| Theta  (4–8 Hz) | 0.808 ±0.210 | 0.872 ±0.216 | *Z*=–2.19, *p=*0.029,  *p corr*=0.145 |  | 0.892 ±0.212 | 0.872 ±0.216 | *Z*=–0.87, *p*=0.387,  *p corr*>0.999 |
| Alpha  (8–12 Hz) | 0.525 ±0.233 | 0.661 ±0.241 | *Z*=–4.18, *p<*0.001,  ***p corr*<0.001** |  | 0.602 ±0.232 | 0.661 ±0.241 | *Z*=–1.58, *p=*0.114,  *p corr*=0.570 |
| Sigma  (12–15 Hz) | 0.224 ±0.214 | 0.387 ±0.247 | *Z*=–5.08, *p<*0.001,  ***p corr*<0.001** |  | 0.280 ±0.215 | 0.387 ±0.247 | *Z*=–3.14, *p=*0.002,  ***p corr*=0.010** |
| Beta  (15–20 Hz) | –0.192 ±0.190 | –0.076 ±0.185 | *Z*=–4.62, *p<*0.001,  ***p corr*<0.001** |  | –0.159 ±0.192 | –0.076 ±0.185 | *Z*=–3.18, *p=*0.001,  ***p corr*=0.005** |
| Data are mean±SD; **^§^**log-transformed spectral power density (log_10_ μV^2^); *p corr*, *p* value after Bonferroni correction (uncorrected *p* value × 5) for correction of multiple comparisons. Values in bold indicate significance after Bonferroni correction (*p* < 0.05). Statistics were performed using Mann Whitney-U test.  Propensity scores were calculated using age group (<50, 50–59, 60–69, and ≥70 years), sex, apnea-hypopnea index (≤15 and >15), alcohol consumption, smoking status, and recent use of medications (TCA, non-TCA, and benzodiazepine).  Abbreviations: NI, non-insomnia; HI, hypnotic user with insomnia complaints; TCA, tricyclic antidepressant; Non-TCA, non-tricyclic antidepressant | | | | | | | |

| **Table S4.** Comparisons of the absolute spectral power density^§^ during NREM and REM sleep between the NI and HI groups before and after propensity score matching | | | | | | | |
| --- | --- | --- | --- | --- | --- | --- | --- |
| *Spectral bands* | *Before propensity score matching* | | |  | *After propensity score matching* | | |
|  | *NI (n=1386)* | *HI (n=65)* | *Statistics* |  | *NI (n=325)* | *HI (n=65)* | *Statistics* |
| NREM |  |  |  |  |  |  |  |
| Delta  (1–4 Hz) | 1.478 ±0.197 | 1.511 ±0.211 | *Z*=–1.61, *p*=0.107,  *p corr*>0.999 |  | 1.478 ±0.197 | 1.462 ±0.207 | *Z*=–1.41, *p*=0.159,  *p corr*>0.999 |
| Theta  (4–8 Hz) | 0.846 ±0.210 | 0.907 ±0.220 | *Z*=–1.23, *p*=0.219,  *p corr*>0.999 |  | 0.808 ±0.210 | 0.872 ±0.216 | *Z*=–0.97, *p*=0.332,  *p corr*>0.999 |
| Alpha  (8–12 Hz) | 0.560 ±0.234 | 0.694 ±0.244 | *Z*=–1.67, *p=*0.049,  *p corr*=0.490 |  | 0.525 ±0.233 | 0.661 ±0.241 | *Z*=–1.65, *p=*0.099,  *p corr*=0.999 |
| Sigma  (12–15 Hz) | 0.258 ±0.218 | 0.420 ±0.256 | *Z*=–4.94, *p<*0.001,  ***p corr<*0.001** |  | 0.224 ±0.214 | 0.387 ±0.247 | *Z*=–3.11, *p=*0.002,  ***p corr*=0.020** |
| Beta  (15–20 Hz) | –0.196 ±0.186 | –0.085 ±0.183 | *Z*=–4.41, *p<*0.001,  ***p corr*<0.001** |  | –0.192 ±0.190 | –0.076 ±0.185 | *Z*=–3.17, *p=*0.002,  ***p corr*=0.020** |
| REM |  |  |  |  |  |  |  |
| Delta  (1–4 Hz) | 1.002 ±0.179 | 1.058 ±0.168 | *Z*=–1.61, *p*=0.107,  *p corr*=0.535 |  | 1.419 ±0.195 | 1.462 ±0.207 | *Z*=–0.91, *p*=0.363,  *p corr*>0.999 |
| Theta  (4–8 Hz) | 0.585 ±0.217 | 0.632 ±0.217 | *Z*=–1.23, *p*=0.219,  *p corr*>0.999 |  | 0.808 ±0.210 | 0.872 ±0.216 | *Z*=–1.23, *p*=0.221,  *p corr*>0.999 |
| Alpha  (8–12 Hz) | 0.319 ±0.239 | 0.405 ±0.239 | *Z*=–2.31, *p=*0.021,  *p corr*=0.105 |  | 0.525 ±0.233 | 0.661 ±0.241 | *Z*=–0.43, *p=*0.665,  *p corr*>0.999 |
| Sigma  (12–15 Hz) | 0.025 ±0.221 | 0.137 ±0.204 | *Z*=–2.19, *p=*0.029,  *p corr*=0.145 |  | 0.224 ±0.214 | 0.387 ±0.247 | *Z*=–1.31, *p=*0.189,  *p corr*>0.999 |
| Beta  (15–20 Hz) | –0.202 ±0.220 | –0.058 ±0.241 | *Z*=–1.97, *p=*0.049,  *p corr*=0.245 |  | –0.192 ±0.190 | –0.076 ±0.185 | *Z*=–1.49, *p=*0.137,  *p corr*>0.999 |
| Data are mean±SD; **^§^**log-transformed spectral power density (log_10_ μV^2^); *p corr*, *p* value after Bonferroni correction (uncorrected *p* value × 10) for correction of multiple comparisons. Values in bold indicate significance after Bonferroni correction (*p* < 0.05). Statistics were performed using Mann Whitney-U test.  Propensity scores were calculated using age group (<50, 50–59, 60–69, and ≥70 years), sex, apnea-hypopnea index (≤15 and >15), alcohol consumption, smoking status, and recent use of medications (TCA, non-TCA, and benzodiazepine).  Abbreviations: NI, non-insomnia; HI, hypnotic user with insomnia complaints; TCA, tricyclic antidepressant; Non-TCA, non-tricyclic antidepressant | | | | | | | |
